# Novel causative variants of VEXAS in *UBA1* detected through whole genome transcriptome sequencing in a large cohort of hematological malignancies

**DOI:** 10.1038/s41375-023-01857-5

**Published:** 2023-02-23

**Authors:** Maki Sakuma, Piers Blombery, Manja Meggendorfer, Claudia Haferlach, Markus Lindauer, Uwe M. Martens, Wolfgang Kern, Torsten Haferlach, Wencke Walter

**Affiliations:** 1 MLL Munich Leukemia Laboratory, Munich, Germany; 2 Medical Graduate Center, Technical University Munich, Munich, Germany; 3 Peter MacCallum Cancer Centre, Melbourne, VIC Australia; 4 Department for Hematology and Oncology, SLK-Clinics Heilbronn, Heilbronn, Germany

**Keywords:** Haematological cancer, Genetics research, Clinical genetics

## Abstract

*UBA1* is an X-linked gene and encodes an ubiquitin-activating enzyme. Three somatic mutations altering the alternative start codon (M41) in *UBA1* in hematopoietic precursor cells have recently been described, resulting in a syndrome of severe inflammation, cytopenias, and the presence of intracellular vacuoles in hematopoietic precursors - termed VEXAS syndrome, a predominantly male disease. Here we present a patient with clinical features of VEXAS who harbored two novel somatic variants in *UBA1* (I894S and N606I). To better understand the clinical relevance and biological consequences of non-M41 (*UBA1*^non-M41^) variants, we analyzed the whole genome and transcriptome data of 4168 patients with hematological malignancies and detected an additional 16 *UBA1*^non-M41^ putative somatic variants with a clear sex-bias in patients with myeloid malignancies. Patients diagnosed with myeloid malignancies carrying *UBA1*^non-M41^ putative somatic variants either had vacuoles or immunodysregulatory symptoms. Analysis of the transcriptome confirmed neutrophil activation in VEXAS patients compared to healthy controls but did not result in a specific transcriptomic signature of *UBA1*^M41^ patients in comparison with MDS patients. In summary, we have described multiple putative novel *UBA1*^non-M41^ variants in patients with various hematological malignancies expanding the genomic spectrum of VEXAS syndrome.

## Introduction

*UBA1* (Ubiquitin-like activating enzyme) is one of the two E1 enzymes of the ubiquitin-proteasome system (UPS) in humans and activates ubiquitins to transfer to E2 enzymes. *UBA1* is located on the X chromosome in humans, but is a known X chromosome inactivation (XCI) escape gene [1, 2] and its total loss of function is considered embryonic lethal in males [3]. In late 2020, Beck et al. described somatic loss of function variants in *UBA1* in the hematopoietic stem and progenitor cell (HSPC) compartment as a cause of a severe hematoinflammatory disease termed VEXAS (Vacuoles, E1 enzyme, X-linked, Autoinflammatory, Somatic) Syndrome [4]. VEXAS, observed exclusively in males, was characterized by treatment-refractory autoinflammatory symptoms, intracellular vacuoles in hematopoietic precursors and cytopenias. Half of the patients with VEXAS were observed to develop myelodysplastic syndromes (MDS) [5]. In the initial description, three causative variants were identified which altered the start codon of the cytoplasmic isoform in exon 3 (M41T, M41L, M41V). Sequencing of *UBA1* exon 3 in more than 1000 patients identified one patient carrying a confirmed somatic non-synonymous variant (S56F), who had milder inflammatory symptoms, vacuoles, and a diagnosis of MDS [6, 7]. The S56F variant was shown to result in partial loss of function. This finding suggests that somatic *UBA1* variants other than those at codon M41 (*UBA1*^non-M41^) may also be relevant in VEXAS syndrome pathogenesis. However, the precise frequency, clinical features and contribution to phenotype for non-M41 variants are unknown.

Herein, we report a patient presenting with the clinical features of VEXAS syndrome who harbored two novel somatic *UBA1*^non-M41^ variants (N606I and I894S) but no *UBA1*^M41^ variant. To further understand the clinicogenomic characteristics of somatic and germline *UBA1*^non-M41^ variants, we analyzed whole genome and transcriptome sequencing data from 4168 patients with hematological malignancies and describe five further *UBA1*^non-M41^ variants as potential novel causes of VEXAS.

## Patients and methods

### Patient cohort

A total of 4168 patients were recruited and analyzed as previously described [8]. The clinical diagnosis was based on standard procedures, following the 2017 WHO classification [9]. The cohort consisted of 774 patients with acute myeloid leukemia (AML), 120 patients with chronic myeloid leukemia (CML), 380 patients with myeloproliferative neoplasms (MPN), 397 patients with myelodysplastic/myeloproliferative neoplasm overlap syndromes (MDS/MPN), which included 78 patients with atypical CML (aCML), 222 patients with chronic myelomonocytic leukemia (CMML), 97 patients myelodysplastic/myeloproliferative neoplasm with ring sideroblasts (MDS/MPN-RS-T), 756 patients with myelodysplastic syndromes (MDS), 321 patients with B-lymphoblastic leukemia/lymphomas (B-ALL), 132 patients with T- lymphoblastic leukemia/lymphoma (T-ALL), 317 patients with chronic lymphocytic leukemia (CLL), 541 patients with mature B-cell neoplasms (labeled B-cell non-Hodgkin lymphoma; B-NHL), which included 97 patients with marginal zone lymphoma (MZL), 94 patients with mantle-cell lymphoma (MCL), 92 patients with hairy cell leukemia (HCL), 68 patients with follicular lymphoma (FL), and 190 patients with other mature B cell neoplasms, 148 patients with mature T- and NK-cell neoplasms (labeled T-cell non-Hodgkin lymphoma; T-NHL), 262 patients with plasma cell myeloma (labeled multiple myeloma; MM), and 20 patients with monoclonal gammopathy of undetermined significance (MGUS). The full details are given as part of Supplementary Table 1. 64 healthy controls were also used for comparison of the transcriptome (35 males and 29 females).

### Whole genome sequencing (WGS) and whole transcriptome sequencing (WTS)

DNA and RNA were extracted from bone marrow and peripheral blood, and libraries were prepared by standard protocols as previously described [10].

### WGS data processing and analysis

Samples were sequenced with an average coverage of 106x. Reads were aligned to the human reference genome (GRCh37, Ensembl annotation) using the Isaac aligner (v3.16.02.19) [11] through BaseSpace’s WGS app (v5, Illumina, San Diego, CA, USA) with default parameters. Tumor-unmatched normal variant calling was performed with a pool of sex-matched DNA (Promega, Madison, WI) using Strelka (v.2.4.7). Variant allele frequency (VAF) of over 2% and a minimum of 5 supporting reads were required for the variants to be reviewed. Variants were queried against the gnomAD database (v.2.1.1) to remove common germline calls (global population frequency >1%) and annotated with Ensembl VEP. Analysis was restricted to protein-altering and canonical splice-site variants. 74 genes and 64 genes were considered driver genes for myeloid and lymphoid malignancies, respectively, as defined in Supplementary Table 2. *UBA1* variants with VAF < 10% were confirmed by targeted sequencing (Supplementary Table 3). For somatic copy number variations (CNV), GATK4 was used following the Broad Institute’s recommended best practices with a panel of normals (defined as patients with a normal karyotype according to conventional cytogenetics).

### Classification of somatic versus germline origin of *UBA1* variants

*UBA1* variants were classified as follows; (i) *putative germline* – present in gnomAD plus VAF over 90% in males or between 40% and 60% in females (ii) *putative somatic* – not present in gnomAD (or present in gnomAD at low allele frequency with VAF supporting somatic origin) plus VAFs outside of the range described as putative germline (iii) *unknown origin* – not meeting criteria above. Frameshift variants not present in gnomAD were considered somatic regardless of their VAF. All putative somatic variants were reviewed for their X chromosome copy number status by standard cytogenetics to consider impact on VAF. When cytogenetic data were unavailable, WGS copy number variation call was referred to. The data necessary for the classification is provided in Supplementary Table 3.

### Transcriptome analysis

Unsupervised clustering was done with uniform manifold approximation and projection (UMAP) using the top 5% most variable genes, excluding genes on the sex chromosomes. Differential gene expression analysis was performed with limma [12] and genes with an adjusted *p*-value < 0.05 and absolute logFC > 1.5 were considered significant. GO enrichment analysis was done by the web-based application Toppfun [13], from where GO gene sets were downloaded (accessed March 15, 2022). Single-sample gene set enrichment analysis (ssGSEA) was done through the GSVA R package [14] with parameter mx.diff = TRUE to suppose upregulation in all members of the gene set. The gene sets were downloaded from MSigDB [15, 16] or taken from references [17, 18] as listed in Supplementary Table 4. All statistical analyses were done using R.

## Results

### Novel somatic *UBA1* variants detected in a patient with clinical features of VEXAS syndrome

A 46-year-old previously healthy male presented with recurrent epistaxis, nodular erythema, increasing fatigue, night sweats and 10 kg of weight loss over several months. The patient denied drug/alcohol use or exposure to toxic substances. Family history was significant for lung cancer affecting his mother, who died at the age of 46. Physical examination revealed pale conjunctiva, jaundice, splenomegaly and erythema nodosum mainly in his trunk. Full blood examination revealed anemia (Hb 5.3 g/dL, reticulocytes 179.4 × 10^3^/µL, MCV 90 fL) and thrombocytopenia (Plt 56 × 10^3^/µL). Indirect bilirubin was high (T-Bil 3.9 mg/dL, D-Bil 0.4 mg/dL), but there were no other signs of hemolysis (LDH 199 U/L, haptoglobin 1.78 g/L). Both iron and ferritin values were low (serum iron 30 µg/dL, ferritin 24.1 µg/L, TS 7.2%), suggestive of iron-deficiency anemia. Hemorrhagic sources other than epistaxis and bleeding disorders besides thrombocytopenia were not identified. CT and gastrointestinal endoscopies showed no tumor or pathologically enlarged lymph nodes, other than enlarged spleen (>25 cm). A bone marrow biopsy was performed which revealed morphological dysplasia and excess blasts (5%), minimal fibrosis and no abnormal lymphoid infiltrate. A diagnosis of MDS-EB1 according to WHO 2017 [9] was made. Abundant vacuoles were observed in hematopoietic precursors and 3% ring sideroblasts were noted. Histological examination of the erythematous rash showed signs of small vessel vasculitis with histiocytes (CD31+, CD4+, MPO+, Lysozyme+, CD68+) and T-cell (CD3+) infiltration. Serological tests for rheumatological diseases including ANCA and ANA were negative. WGS was performed as part of the SIRIUS study (clinicalTrials.gov Identifier: NCT05046444) on a bone marrow aspirate sample which revealed two variants in *UBA1 -* I894S (VAF 56%) and N606I (VAF 9%). Neither of these variants was detected in CD3+ cells isolated from peripheral blood from the same patient consistent with their somatic origin. No typical MDS driver variants were detected.

### Putative somatic *UBA1*^non-M41^ variants are detectable across hematological malignancies with a clear sex-bias in myeloid malignancies

**Fig. 1:**
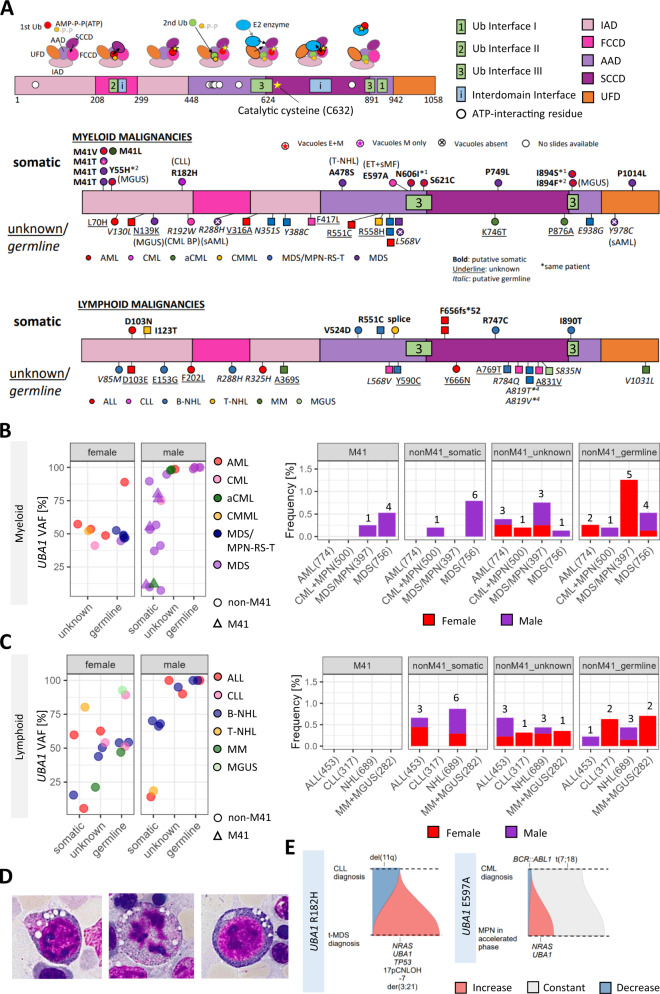
Distribution of variants in the gene *UBA1* and their relevance in hematological malignancies. **A** (Above) The gene *UBA1* consists of five functional domains: two adenylation domains (active adenlyaltion domain [AAD] and inactive adenylation domain [IAD]), two catalytic half domains (first catalytic cysteine half domain [FCCD] and second catalytic cysteine half domain [SCCD]), and ubiquitin fold domain [UFD]. UBA1 IAD and AAD adenylate the first ubiquitin, and transfer ubiquitin to the catalytic cysteine domains to form a thioester bond. The second ubiquitin needs to be loaded to make the necessary conformational changes to transfer the charged ubiquitin to E2 enzymes. The regions constituting the interface contacting ubiquitin (Ub Interface I to III) defined in references [19] and [20] are labelled as boxes. Selected domains important in conformational changes are also represented in boxes. ATP-interacting residues mentioned in the Supplementary Fig. S12 of Beck et al. [4] are shown as circles. (Below) Loci of the variants are shown, separated by whether the patient was diagnosed with myeloid or lymphoid malignancies. *(number) denotes that the variants are carried by the same numbered patient. The putative somatic variants (bold) are shown above and putative germline (italic) and variants of unknown significance (underline) are shown below the gene. The presence of vacuoles are shown by signs inside the circle (male) or square (female). No signs means that bone marrow smear slides were not preserved at the facility. E + M: vacuoles both in erythroids and myeloids. M only: vacuoles in myeloids only. The diagnosis are color coded and the second diagnosis, if present, are denoted in brackets. AML (acute myeloid leukemia), CML (chronic myeloid leukemia), aCML (atypical chronic myeloid leukemia), CMML (chronic myelomonocytic leukemia), MDS/MPN-RS-T (myelodysplastic/myeloproliferative neoplasm with ring sideroblast and thrombocytosis). ALL (acute lymphoblastic leukemia), CLL (chronic lymphoblastic leukemia), B-NHL (B-cell non-Hodgkin lymphoma), T-NHL (T-cell non-Hodgkin lymphoma), MM (multiple myeloma), MGUS (monoclonal gammopathy of uncertain significance), CML BP (blast phase), sAML (secondary AML), ET (essential thrombocythemia), and sMF (secondary myelofibrosis). (B-C) Distribution of variant allele frequency by the lineage of the malignancies, and the frequency of each diagnostic categories and distribution of sexes are shown. The number of patients in the analyzed cohorts are shown in brackets. Further details are given in Supplementary Table 1. **B** Myeloid malignancies. **C** Lymphoid malignancies. **D** Characteristic vacuoles were present in myeloid precursors, even in mitotic phase, and in erythroid precursors from bone marrow aspirates (400x, oil) of the patient harboring variants Y55H and I894F. **E** Likely clonal history of two patients with longitudinal data available are shown.

Given the identification of potential novel *UBA1* variants causing VEXAS syndrome outside of the M41 codon, we went on to analyze WGTS of 4168 patients (see “Material and Methods”) with a broad range of myeloid and lymphoid malignancies. From this testing we identified 5 patients with *UBA1*^M41^ variants, 16 patients with putative somatic *UBA1*^non-M41^ variants, 20 patients with putative germline *UBA1*^non-M41^ variants and 16 patients where the origin of the *UBA1* variant was not able to be definitively assigned. Three patients had longitudinal data available to confirm somatic status in two (R182H and E597A) and in one patients harboring a putative germline variant (R192W), no change in VAF (100%) was observed. Whilst identified variants were distributed over the whole coding region of *UBA1*, only putative somatic variants were observed in Ubiquitin interface III domain of the protein [19, 20] (Fig. 1A). The VAF ranged from 5% to 89% for the putative somatic variants, 21% to 100% for the unknown variants, and 44% to 100% for the putative germline variants, as certain variants with somatic VAFs are classified non-somatic due to their X chromosomal copy number status. Interestingly, a clear sex bias could be detected in myeloid malignancies, as potential somatic variants occurred only in males (Fig. 1B), whereas no sex difference was observed in occurring in lymphoid malignancies (Fig. 1C). The genetic and clinical characteristics of all the 12 patients with myeloid malignancies carrying the putative somatic variants are shown in Table 1.

**Table 1:** Genetic and clinical characteristics of the 12 patients with myeoid malignancies carrying putative somatic *UBA1* variants.

Patient_ID	Age	Sex	Diagnosis	*UBA1* Variant	VAF [%]	Vacuole	Co-mutation	Karyotype	Hematological history (age)	Possible immunodysregulation (age)
P1	77	male	aCML	M41L	12	NA	*NRAS* (12%)	46,XY[20]	clinically diagnosed with CML (65); CML diagnosis revised to aCML due to negative *BCR::ABL1* [*](75); splenomegaly (circa 40 cm)	none recorded
P2	82	male	MDS-MLD	M41T	76	NA	none	46,XY[20]	ESA/transfusion-requring anemia	none recorded
P3	76	male	MSD-RS-SLD	M41T	11	yes	*SF3B1* (31%), *JAK2* (14%), *ZRSR2* (4%)	46,XY[20]	thrombocytosis	none recorded
P4	50	male	MDS-MLD	M41T	80	NA	none	46,XY,t(2;11)(p21;q23)[13]/46,XY [7]	NA	unspecified autoimmune disease under immunosuppresion
P5	84	male	MDS-MLD	M41V	55	yes	none	46,XY,der(1)t(1;2)(p34;q24),der(2)t(1;2)(p36;q24)[7]/46,idem,del5(q14q34)[3]/46,XY[10]	transfusion-requring anemia, pancytopenia	polyneuropathy, latent hyperthyroidism
P6	76	male	MDS-RS-MLD	Y55H	41	yes	*SRSF2* (50%)	46,XY[20]	[*]MGUS; pancytopenia; anemia treated with 30 mg prednisone/transfusion, successfully discontinued (79)	none recorded
				I894F	37					
P7	66	male	CLL	R182H	0	yes		46,XY, del(11)(q21q23)[4]/46,XY[16]	[*]CLL treated with fludarabine, cyclophosphamide, rituximab (64-65) in CR (66); pancytopenia(65); treated with azacitidine (66)	Pneumocystis pneumonia (65/66), CMV reactivation (65/66)
			t-MDS/MDS-EB2		57		*NRAS* (35%), *TP53* (98%)	44,XY,der(3;21)(q10;q10),-7[20]		
P8	68	male	MDS-MLD	A478S	53	NA	none	45,X,-Y [18]/46,XY[2]	primary cutaneous large cell anaplastic T cell lymphoma in the head and neck treated with resection [*] and radiation (68-70); MDS no treatment until age 70	hyperthyroidism
P9	69	male	CML	E597A	0	NA	*BCR::ABL1* (IS 0.034)	46,XY,t(7;18)(q11,q23)[7]/46,XY[2]	ET (61) treated with anagrelide; CML [*1] treated with imatinib (67); anemia due to secondary MF (69); transfusion-dependent Coombs-positive anemia (69); 12% blasts with increased borderline monocyte/blasts in PB [*2] (69)	monoblast infiltration in the skin (69), steroid-resistant fever (69)
			MPN		75		*NRAS* (50%), *BCR::ABL1* (IS 0.006)	46,XY,t(7;18)(q11,q23)[20]		
P10	67	male	MDS-RS-MLD	S621C	90	yes	*FLT3*-ITD (39%), *RUNX1* (69%), *SRSF2* (39%)	47,XY, + 21[19]/46,XY[1]	steroid-treated hemolytic anemia[*] (67); 9 cycles of azacitidine against MDS (67); decitabine switch (68), 33% blasts in PB, suggesting secondary AML (68); transfusion-dependent anemia	azacitidine injection site reaction (67), antibiotic-resistant broncoscopy-negative pulmonary infiltrate post fungal infection (68)
P11	73	male	MDS-MLD	P749L	10	NA	none	47,XX, + X[20]	chronic hemolytic anemia requiring ESA/transfusion	hyperthyroidism treated with nuclear medicine (74)
P12	63	male	MDS-RS-SLD	P1014L/canonical splice site	7	NA	*SF3B1* (49%), *TET2* (16%)	46,XY[20]	autoimmune anemia treated with steroids (62-, tapered and reinitiated), azathioprine[*] (62–63), mycophenol-mofetil (63–74), and rituximab (63, 74), ESA/multiple transfusion (62-)	anti-parietal cell antibody positive gastritis with polyclonal hypergammaglobulinemia and elevated complements (56), IgG4-associated nephritis (58/histology 68), interstitial pneumotitis (62), leiomyosarcoma (71), diverse skin tumors (74)

[*] indicates the time point the sequencing was performed.

*MDS-MLD* MDS with multi-lineage dysplasia, *MDS-RS-MLD* MDS with ring sideroblasts and multi-lineage dysplasia, *CLL* chronic lymphocytic leukemia, *t-MDS* therapy-related MDS, *MDS-EB* MDS with excess blast, *CML* chronic myeloid leukemia, *MPN* myeloproliferative neoplasms, *MDS-RS-SLD* MDS with ring sideroblasts and single-lineage dysplasia, *aCML* atypical CML, *ESA* Erythropoiesis-stimulating agent, *NA* not available, *MGUS* monoclonal gammopathy of undetermined significance, *CR* complete remission, *ET* essential thrombocythemia, *MF* myelofibrosis, *PB* peripheral blood, *AML* acute myeloid leukemia.

Consistent with previous descriptions [4], *UBA1*^M41^ variants occurred exclusively in males in our cohort and in myeloid malignancies (MDS *n* = 4, MDS/MPN [aCML] *n* = 1, Fig. 1B) but no *UBA1*^M41^ variant was detected in patients with AML. For 2/4 MDS patients with *UBA1*^M41^ variants, material was available for morphological assessment, which confirmed the presence of vacuoles in hematopoietic precursors.

Sixteen putative somatic *UBA1*^non-M41^ variants were detected in 16 patients across both myeloid and lymphoid malignancies (Fig. 1B, C). *UBA1*^non-M41^ variants were observed exclusively in males in patients with myeloid malignancies (MDS *n* = 6, CML + MPN [ET + CML + sMF] *n* = 1), with 8 *UBA1*^non-M41^ variants observed in 7 patients (Table 1). Three patients with MDS and *UBA1*^non-M41^ variants (Y55H + I894F, R182H, S621C) had material available for morphological assessment of which all three showed vacuoles. Images of a representative patient (Y55H + I894F) are shown in Fig. 1D and Supplementary Fig. 1. In lymphoid malignancies, the distribution of putative somatic *UBA1*^non-M41^ across sexes was essentially equal with nine patients (5 male, 4 female) found to carry *UBA1*^non-M41^ putative somatic variants (B-ALL *n* = 3, B-NHL *n* = 4 [FL *n* = 3, HCL *n* = 1], T-NHL *n* = 2, Fig. 1A). Notably the same frameshift variant (F656SfsTer52) was identified in two female patients with ALL.

Concerning the *UBA1* unknown origin and putative germline variants, the incidence showed a slight female predominance with M:F ratios of 1:1.3 and 1:1.6, respectively (total cohort M:F ratio 1:0.7). No patients carrying putative somatic variants were diagnosed with MDS/MPN-RS-T, however, 5 patients carrying putative germline variants were diagnosed with MDS/MPN-RS-T, all of whom were female (MDS/MPN-RS-T cohort male to female ratio = 1:1.1). Material was available for morphological assessment in 3 patients with putative germline origin *UBA1* variants. None of these three patients (R288H, Y978C, and L568V) had vacuoles present.

### Immunodysregulatory symptoms are common among patients with myeloid malignancies carrying *UBA1*^non-M41^ variants

If available, non-hematological co-diagnoses and history of patients, as provided by the referring physician, were extracted from the request form. The records of the 57 patients carrying *UBA1* variants were examined, with particular attention to symptoms suggestive of VEXAS in the 16 patients carrying the putative somatic *UBA1*^non-M41^ variants. Whilst the records were incomplete, among the patients with putative somatic variants, 5 out of the 7 patients with myeloid malignancies had immunodysregulatory symptoms documented, whereas no unprovoked inflammatory symptoms were noted for the 9 patients with lymphoid malignancies. Table 1 summarizes the results of the 7 patients with myeloid malignancy carrying the putative somatic *UBA1*^non-M41^ variants along with the record of the 5 patients carrying the *UBA1*^M41^ variants. The documented clinical information included IgG4-associated nephritis/pneumonitis (P1014L, simultaneously affecting the canonical splice site), skin monoblast infiltration (E597A), hyperthyroidism (A478S and P749L), Coombs-positive hemolytic anemia (E597A), and sterile pulmonary infiltrate (S621C). Of interest, two patients developed asynchronous malignancies in the skin of the head and neck area after they were discovered to carry *UBA1* likely somatic variants. The patient carrying the A478S variant was diagnosed with primary cutaneous anaplastic large-cell lymphoma of both ears, left temporal and right occipital regions recurring in a period of two years. The patient carrying the P1014L variant was under immunosuppressive treatment, when he developed basosquamous carcinoma (right nasolabial and cheek), squamous cell carcinoma (right retroauricular region), and Bowen’s disease (right cervical region) within a period of two months.

Among the patients with *UBA1* variants of unknown origin or putative germline, Graves’ disease with recurrent thyroid nodules development after goiter operation was documented for one MDS patient (L568V) and there were two additional patients with thyroid involvement (hypothyroidism, R784Q, CLL; intrathyroidal parathyroid carcinoma, N139K, MDS). Polyarthritis (Y978C, MDS), childhood tonsillectomy (L568V, MDS/MPN-RS-T), steroid-treated sinusitis, pneumonitis, and non-malignant renal mass (Y388C, MDS/MPN-RS-T), and unclear polyneuropathy before chemotherapy (R551C, AML) were also documented.

### The majority of patients with *UBA1* putative somatic variants in myeloid malignancies have co-mutations in leukemic driver genes

In order to understand the broader genomic context of putative somatic *UBA1* variants in myeloid malignancies, we searched for chromosomal aberrations and co-mutations in known leukemia driver genes (Table 1). Various co-mutations were detected in 7/12 (58%) patients, with *NRAS* (*n* = 3), *SF3B1* (*n* = 2) and *SRSF2* (*n* = 2) being the most frequent. The identified leukemogenic driver variants were present at higher VAFs than the X-chromosome adjusted VAF of *UBA1* variants and, hence, these *UBA1* clones are likely sub-clonal to the driver clones by pigeon-hole principle, assuming heterozygosity of the driver variants. Among the patients harboring *NRAS* variants, two carrying the *UBA1* variants R182H (MDS) and E597A (CML + secondary myelofibrosis [MF] after essential thrombocythemia [ET] treatment) were known with a history of prior treatment against CLL (rituximab-fludarabine-cyclophosphamide; R-FC) and CML (imatinib), respectively. For both cases, WGS data were available at the pretreatment time point, which showed that the *UBA1* and *NRAS* clones occurred only after treatment (Fig. 1E). In 2 patients (P4, M41T with t(2;11); P5, M41V with del(5q)), disease-defining chromosomal aberrations were noted to be at a smaller clone size than the *UBA1* variant clones. In 3 patients (M41T, P749L, A478S) no apparent leukemogenic driver variants or disease-defining chromosomal aberrations were noted.

### Whole transcriptome analysis of patients with *UBA1*^non-M41^ variants

**Fig. 2:**
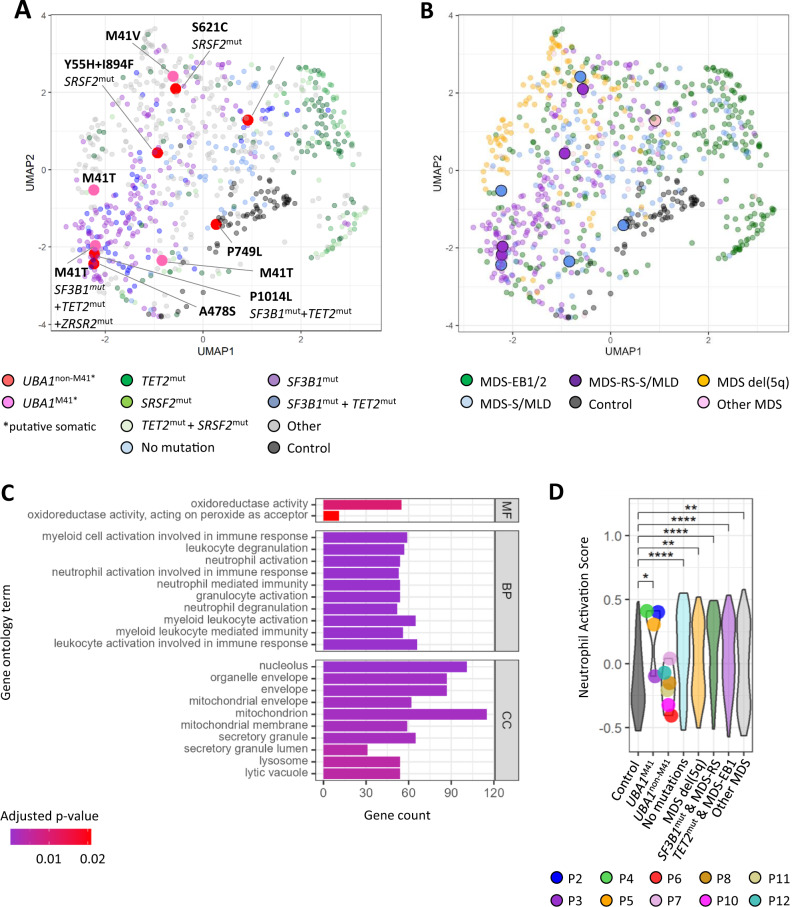
Transcriptomic analysis of patients carrying putative somatic *UBA1* variants in comparison to other MDS patients. **A** UMAP of 816 (752 MDS patients, 64 healthy controls) samples color-coded based on selected co-mutations. **B** UMAP of 816 (752 MDS patients, 64 healthy controls) samples color-coded based on WHO sub-classification. MDS-EB (MDS with excess blast), MDS del5q (MDS with isolated del(5q)), MDS-RS (MDS with ring sideroblasts), MDS-MLD (MDS with multi-lineage dysplasia), MDS-SLD (MDS with single-lineage dysplasia) (**C**) GO analysis of upregulated genes of *UBA1*^M41^ patients compared to healthy controls. **D** ssGSEA (single-sample gene set enrichment analysis) scoring of neutrophil activation stratified by selected groups. Wilcoxon rank sum test was used with Benjamini & Hochberg correction. *p < 0.05, **p < 0.01, ***p < 0.001, ****p < 0.0001.

To better understand the biological effect and inflammogenicity of somatic *UBA1*^non-M41^ variants we analyzed the transcriptome from patients diagnosed with MDS. The samples were taken at the time of their first diagnosis of MDS, and the disease severity and treatment status of their co-existing non-hematological conditions, if any, were not uniformly available. An initial unsupervised clustering analysis based on the top 5% most variable genes (1012 genes) showed that samples with putative somatic *UBA1* variants did not form a distinct group (Fig. 2A, B). As the top 5% most variable genes did not capture the characteristics of *UBA1*^M41^ samples, we subsequently performed supervised differential gene expression (DEG) analysis. Comparing the transcriptional profiles of *UBA1*^M41^ samples with healthy controls resulted in 1038 DEGs with 906 genes up-regulated and 132 genes down-regulated, respectively. GO enrichment analysis of up-regulated genes in *UBA1*^M41^ patients revealed an enrichment of myeloid cell/neutrophil activation and degranulation, as reported by Beck et al. [4] for VEXAS patients (Fig. 2C). To evaluate *UBA1*^non-M41^ variants individually in the context of VEXAS-upregulated neutrophil activation we analyzed the samples applying the single-sample gene set enrichment analysis method [14], which ranks and assigns an enrichment score of gene sets to each sample within the group. We created a group of MDS patients confirmed not to have any driver mutations (VAF > 2%) or chromosomal abnormalities to simulate background MDS controls as well as MDS subgroups reported with positive (del5q [21], *TET2*^mut^/MDS-EB1 [22, 23]) or negative (*SF3B1*^mut^/MDS-RS [22, 23]) association to inflammation. High neutrophil activation scores were not specific to *UBA1*^M41^ samples, and some MDS samples carrying no leukemia-driver mutations scored over top quantile (Fig. 2D). None of the *UBA1*^non-M41^ samples had reached over top quantile scores. Results of other myeloid cell/neutrophil activation and degranulation gene sets did not differ (data not shown).

To complement the functional analysis, we evaluated pathways that may be involved in VEXAS inflammation in a hypothesis-driven manner. Briefly, we selected pathways targeted by anti-inflammatory treatment given to VEXAS patients [24–28]. Furthermore we examined pathways related to autoinflammatory diseases due to loss of function variants in the proteasome genes [29–31], which are expected to provide mechanistic insights into VEXAS inflammation [32], as they both may induce interferon response via the unfolded protein response (UPR) pathway. *UBA1*^M41^ samples had significantly higher scores of the Interferon response gene set (IRG-6) [18], but did not differ to other MDS subgroups (Supplementary Fig. 2). Thus, *UBA1*^M41^ samples did not show any specific and sensitive inflammatory signature.

## Discussion

*UBA1* is a newly rediscovered gene in the field of hematology in the context of VEXAS syndrome, a prototypical hematoinflammatory disease. By analyzing comprehensive and unbiased WGTS data from 4168 patients we identified five *UBA1*^M41^ variants (total cohort 0.1%; myeloid malignancy cohort 0.2%; MDS cohort 0.5%), 16 *UBA1*^non-M41^ putative somatic variants (total cohort 0.4%), including eight *UBA1*^non-M41^ variants in seven patients with myeloid malignancies (myeloid malignancy cohort 0.3%; MDS cohort 0.8%), representing potential causative variants of VEXAS syndrome. In addition, nine *UBA1*^non-M41^ variants in patients with lymphoid malignancies (lymphoid malignancy cohort 0.5%) were detected. The incidence of *UBA1* somatic variants in a community cohort has recently been reported to be approximately 1 in 4000 of older males [33]. The relatively high incidence of somatic *UBA1* variants in our cohort of hematological malignancy suggests that *UBA1* variants are not passenger variants. We further detected a clear sex bias in the occurrence of *UBA1* somatic variants in myeloid malignancies, which is supportive of the potential clonal advantage conferred by *UBA1* variants. In these male patients of myeloid malignancies, we observed three different scenarios: (i) *UBA1* variants as the main clone, (ii) *UBA1* variants as subclonal events in combination with known leukemic driver events, and (iii) *UBA1* variants as a secondary major driver event following treatment. Scenario (iii) has been described in one case report of a patient initially diagnosed with ET treated with hydroxyurea subsequently developing MDS after acquiring a *UBA1* variant [34]. Of note two of our confirmed somatic variants (R182H, E597A) co-emerged with a *NRAS* variant after treatment (R-FC for CLL + t-MDS R182H; imatinib for ET + CML + sMF E597A), which again suggests the contribution of *UBA1* variants to clonal fitness. Among the variants we identified, P749L is a known partial loss of function mutation [35, 36]. Some of the identified somatic variants were also on functional residues (S478, R551, N606, S621, R747), one of which is a partial loss of function site (R747). The literature and prevalence in public databases on the identified putative somatic variants are summarized in Table 2 [7, 20, 33, 35–44]. Frameshift variants, which are likely total loss of function, were only discovered in females (F656SfsTer52), which may also be partial loss of function overall considering the two copies of the X chromosome. None of the identified variants in males were on total loss of function sites, but only proximal to such sites (V524D, E597A). Experimentally, partial loss of function mutations are known to result in a proliferative phenotype, whereas total loss of function mutations results in an apoptotic phenotype [35, 36]. Thus, it is possible that variants leading to partial loss of UBA1 function contribute to the development of cancers.

**Table 2:** Literature review on the identified putative somatic missense variants in the hematological malignancy cohorts.

Patient ID	Cluster	Diagnosis	Variant	Cohort freq^1^	Public freq^2^	Proximal functional site	Methods	Function/mutant consequence/mutant phenotype	Reference	Organism
P6	NA	MDS	Y55H	1/756; 0.0013	0	S56F	mutagenesis	temperature-sensitive partial loss of function	Poulter [7]	human
						R57	structural	ATP-contacting residue (gamma phosphate)	Lv [20]	human
						R57K/A	mutagenesis	significantly reduced nitrate reductase activity in R515 (R73 in E. Coli) double mutants	Lake [40]	E. Coli (R14K/A)
P33	NA	B-ALL	D103N	1/321; 0.0031	0	S140	structural	SCCH/IAD/AAD interacting residue; substitution has minimal effect on thioester bond formation	Hann [41]	S. Pombe (Q105A)
P40	NA	T-NHL	I123T	1/148; 0.0068	0	S140	structural	SCCH/IAD/AAD interacting residue; substitution has minimal effect on thioester bond formation	Hann [41]	S. Pombe (Q105A)
P7	NA	MDS	R182H	1/756; 0.0013	gnomAD (3/165293; 1.81e-5)	A189T*	mutagenesis	slightly less efficient ubiquitination, higher apoptotic rate than wild type	Lao [42]	mouse (A189T)
P8	ATP-binding	MDS + T-NHL	A478S	1/756; 0.0013	0	A478	structural	ATP-contacting residue (alpha phosphate); consists the oxyanion hole and GxGxxG ATP binding motif	Lv [20]	human
P37	ATP-binding	B-NHL (FL)	V524D	1/541; 0.0018	0	K528N*	mutagenesis	mutant tissue apoptotic and enlarged wild type tissue (total loss of function)	Pfleger [39]	Drosophila(K663N)
						K528A	structural; mutagenesis	ATP-contacting residue (beta phosphate); ATP binding attenuated and reduced Ub-adenylation	Tokgöz [43]	human
P39	ATP-binding	B-NHL (HCL)	R551C	1/541; 0.0018	0	N550*	mutagenesis	3-fold decrease in thioester bond formation	Hann [41]	S. Pombe (E511R)
						R551	structural	ATP-contacting residue	Lv [20]	human
P9	Ub Interface III	CML + MPN	E597A	1/500; 0.0020	0	C588Y*	mutagenesis	mutant tissue apoptotic and enlarged wild type tissue (total loss of function)	Pfleger [39]	Drosophila (C723Y)
						G599	structural	ubiquitin-contacting residue	Lv [20]	human
case	Ub Interface III	MDS	N606I	1/756; 0.0013	0	N606	structural	van der Waals contacts with Ub	Lv [20]	human
P10	Ub Interface III	MDS	S621C	1/756; 0.0013	COSMIC MDS-RS (1/9; 0.11)	S621	structural	hydrogen bond with Ub	Lv [20]	human
						S621C	sequencing	somatic variant identified in a *SF3B1*-mut MDS patient (COSMIC-cited)	Papaemmanuil [44]	human
						S621C	sequencing	somatic variant identified in a patient with vasculitis	Beck [36]	human
P36	Interdomain Interface	B-NHL (FL)	R747C	1/541; 0.0018	COSMIC colorectal carcinoma (1/619; 0.0016)	R747E*	mutagenesis	4-fold decrease in thioester bond formation	Hann [41]	S. Pomne (R707E)
						R747	structural	salt bridge between E252 of the FCCH domain	Lv [20]	human
						P747C	sequencing	somatic variant identified in a female patient with large intestine adenocarcinoma (COSMIC-cited)	Giannakis ()	human
P11	Interdomain Interface	MDS	P749L	1/756; 0.0013	0	P749L*	mutagenesis	enlarged mutant tissue (temperature-sensitive partial loss of function)	Pfleger [39], Lee [38]	Drosophila (P884L)
P38	Ub Interface III	B-NHL (FL)	I890T	1/541; 0.0018	0	I891	structural	van der Waals contacts with Ub	Lv [20]	human
P6	Ub Interface III	MDS	I894F	1/756; 0.0013	0	I891	structural	van der Waals contacts with Ub	Lv [20]	human
case	Ub Interface III	MDS	I894S	1/756; 0.0013	0	I891	structural	van der Waals contacts with Ub	Lv [20]	human
P12	NA	MDS	P1014L/splice	1/756; 0.0013	0	R1010	structural	E2 enzyme (Ubc4) contact site	Olsen [45]	S. Pombe (R965)
						del(923-1058)	mutagenesis	IRF3 binding lost	Chen [46]	human/zebrafish

[*] indicates that the site is a corresponding conserved residue in human.

1: The frequency in the respective subcohort.

2: The frequency reported in gnomAD or COSMIC databases. In case of COSMIC the cancer name is given and the frequency is in the brackets.

Subject Ontology:UBA1, VEXAS, MDS, inflammation, cytopenia, leukemia, lymphoma, sex difference.

Whilst there are no official diagnostic criteria for VEXAS syndrome, VEXAS is characterized by the co-existence of acquired inflammatory and hematological symptoms as well as the presence of vacuoles in hematopoietic stem and progenitor cells and somatic *UBA1* mutations [4, 24]. Our index case showed all the clinical features of VEXAS but carried two novel *UBA1*^non-M41^ variants (N606I VAF 9%, I894S VAF 56%) instead of the canonical *UBA1*^M41^ variant, one of which variant locus was identified in another patient with double mutant (Y55H VAF 41%, I894F VAF 37%) in our cohort. The repeated appearance of the I894 loci and the high VAF suggests I894 as a potential disease-causative loci of VEXAS. Interestingly, residues N606 and I894 face each other at the ubiquitin interface III, and the likely later acquisition of N606 with a lower VAF may have had an advantageous effect for clonal survival. We additionally report that 5 of the 7 patients diagnosed with myeloid malignancies in the retrospective cohort carrying somatic *UBA1*^non-M41^ variants (A478S, E597, S621C, P749L, P1014L) showed immunodysregulatory symptoms (Table 1), although a clinical rheumatologic diagnosis was recorded in only one variant (P1014L). Undifferentiated inflammatory symptoms are frequently observed in patients harboring *UBA1*^M41^ mutations [6, 28, 45, 46], and our records on patients carrying *UBA1*^non-M41^ variants would contribute to the shaping of VEXAS Syndrome as a spectrum of inflammatory manifestations.

As the uniqueness and pathogenesis of *UBA1*^M41^ somatic variants in hematopoietic precursors pertains to its inflammogenicity, we analyzed the transcriptome with respect to inflammation-related signatures. However, no *UBA1*^M41^-specific signature could be identified. Furthermore, activation of inflammatory pathways relevant in VEXAS syndrome were neither specific nor sensitive in comparison to other patients with MDS. The retrospective nature of the dataset did not allow us to assign the state of active inflammation or reception of anti-inflammatory treatment, leaving the possibility that specific VEXAS inflammation is detectable when patients at disease onset are compared. However, 10–20% of patients with MDS show treatment-refractory systemic autoimmune and inflammatory diseases [22] with *UBA1*^M41^ variants only identifiable in the minority [47, 48]. Moreover, since not only autoinflammatory (dysregulation of innate immunity) but also autoimmune diseases (dysregulation of acquired immunity/antibody-mediated) can be part of VEXAS presentation [49], it may be that a transcriptomic signature specific for the inflammation seen in VEXAS does not exist. This necessitates the emphasis of *UBA1* somatic mutations in the definition of VEXAS rather than defining the disease based on the co-existence of inflammatory symptoms and cytopenias, which can be observed in patients with MDS harboring other mutations [23, 50, 51] and chromosomal abnormalities [52, 53].

Although only anecdotal, an intriguing finding in our cohort are the described recurrent malignancies in the skin for two MDS patients carrying putative *UBA1*^non-M41^ somatic variants. Skin cancer (Merkel cell carcinoma) has recently been reported in one CMML patient carrying a S56F variant [48]. In addition to being apoptotic, *UBA1* total loss of function variants are known to para-clonally cause an overgrowth of the surrounding wild type tissues [35, 36]. Moreover, *UBA1*^M41^ variants were reported to lead to para-clonal cutaneous involvements in VEXAS syndrome [54]. Thus, non-hematological co-malignancies may also have relevance to the presence of *UBA1* variants by para-clonal effect.

Because of the retrospective nature of our study and the associated incompleteness of the clinical records, our work can only provide a limited assessment of the clinical relevance of *UBA1*^non-M41^ variants. In addition, we did not have the option to investigate matched germline material to definitively assign somatic and germline status. As VEXAS pertains to somatic variants, we adopted a conservative threshold of 90% for males to assign somatic status. However *UBA1*^M41^ somatic variants in VEXAS patients may show exceedingly high VAF (>90%) [55, 56], and the *UBA1*^non-M41^ variants classified as unknown and putative germlines may well have been somatic. Furthermore, it should be noted that rare germline variants are sometimes found as somatic variants in MDS/AML patients [57, 58], and the assignment of germline origin does not exclude its potential pathogenicity or predisposition to the disease. One of the identified variants (R551C) was classified as putative somatic in a patient with T-NHL, whereas it was classified as unknown in a patient with AML, and the classification approach is limited in understanding the effect of the variant on the function of the gene. Finally, the transcriptomic analysis was limited because of the relatively small number of patients with *UBA1* putative somatic variants in our cohort.

In summary, by applying comprehensive WGTS data analyses we identified several potentially clinically relevant *UBA1*^non-M41^ variants that contribute to a more detailed and exhaustive description of the landscape of *UBA1* variants in hematologic malignancies. Further functional studies of these novel *UBA1* variants are warranted to understand their contribution to the VEXAS phenotype. However, given the broad spectrum of identified variants, we recommend that future VEXAS studies should include the entire *UBA1* gene for analysis in order to better understand the variants causing this rare inflammatory syndrome.

## Supplementary information

Supplementary Table 1

Supplementary Table 2

Supplementary Table 3

Supplementary Table 4

Supplementary Figure Legends

Supplementary Figure 1

Supplementary Figure 2

## Data Availability

The datasets generated during and/or analysed during the current study are available from the corresponding author on reasonable request.
