# Supplementary material for: Novel causative variants of VEXAS in UBA1 detected through whole genome transcriptome sequencing in a large cohort of hematological malignancies
Source: Leukemia. 2023 Feb 23;37(5):1080–91. doi: 10.1038/s41375-023-01857-5 (PMC10169658; doi:10.1038/s41375-023-01857-5)
Supplement: Supplementary file 5 — Supplementary Figure Legends [file 41375_2023_1857_MOESM5_ESM.docx]

**Supplementary Figure 1. Vacuoles of the patient harboring *UBA1* variants Y55H and I894F**

Bone marrow aspirate (400x, oil) showing characteristic vacuoles present in myeloid (orange arrows), and erythroid (red arrows) precursors.

**Supplementary Figure 2. Inflammatory pathway analysis.**

(A) Simplified representation of the inflammatory pathways analyzed. The gene sets were taken from MSigDB or references as listed in Supplementary Table 4. (B) ssGSEA (single-sample gene set enrichment analysis) scoring of each gene sets stratified by selected groups. Each plot shows the enrichment scores of the gene sets as numbered in (A). Samples carrying *UBA1*^M41^ and *UBA1*^non-M41^ variants are represented in dots and color coded in addition to its representation as violin plots. Wilcoxon rank sum test was used with Benjamini & Hochberg correction. *p < 0.05, **p < 0.01, ***p < 0.001, ****p < 0.0001.
