# Supplementary figures and images for: Novel causative variants of VEXAS in UBA1 detected through whole genome transcriptome sequencing in a large cohort of hematological malignancies
Source: Leukemia. 2023 Feb 23;37(5):1080–91. doi: 10.1038/s41375-023-01857-5 (PMC10169658; doi:10.1038/s41375-023-01857-5)

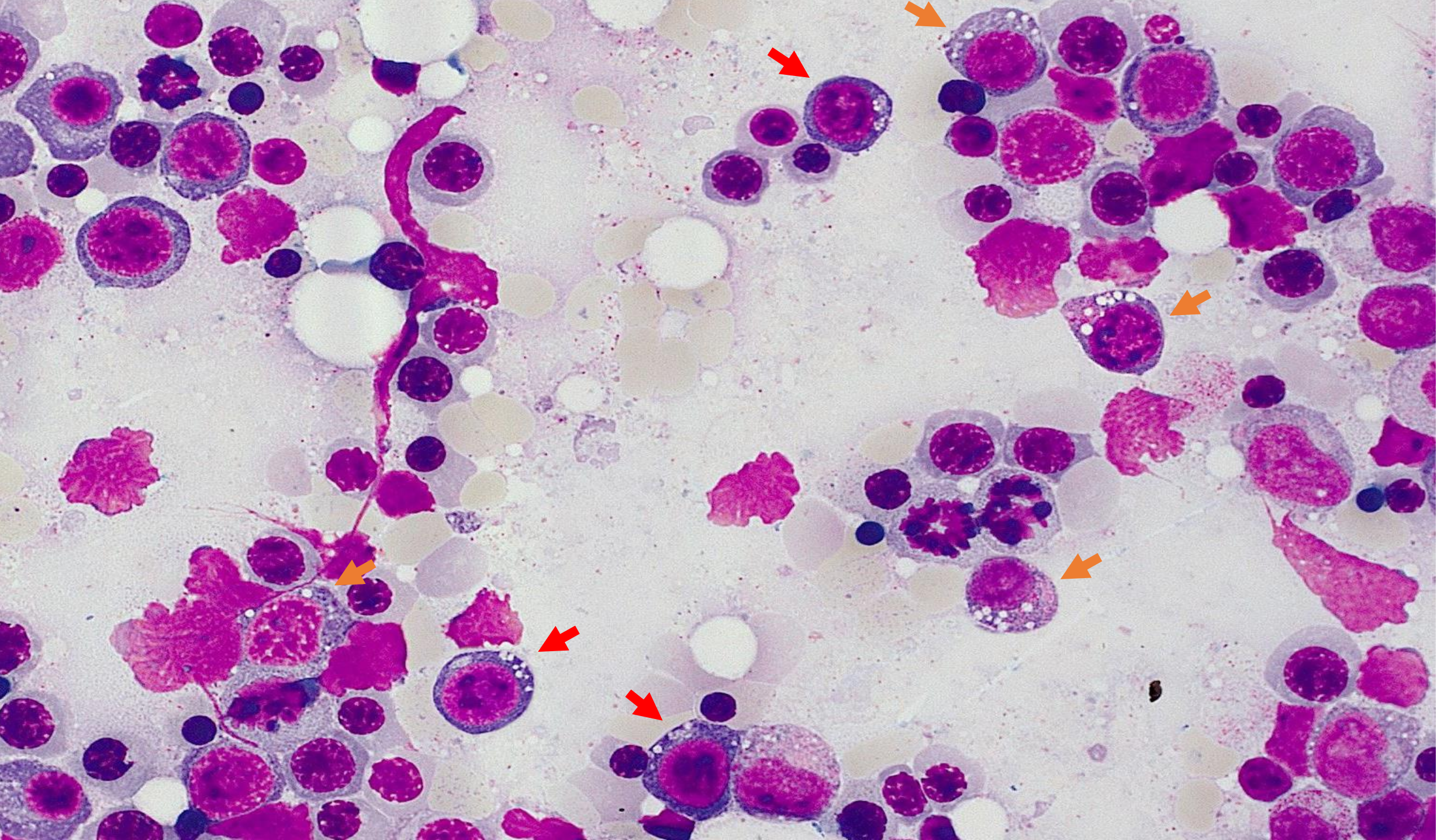

Supplement: Supplementary file 6 — Supplementary Figure 1 [file 41375_2023_1857_MOESM6_ESM.pdf]

A

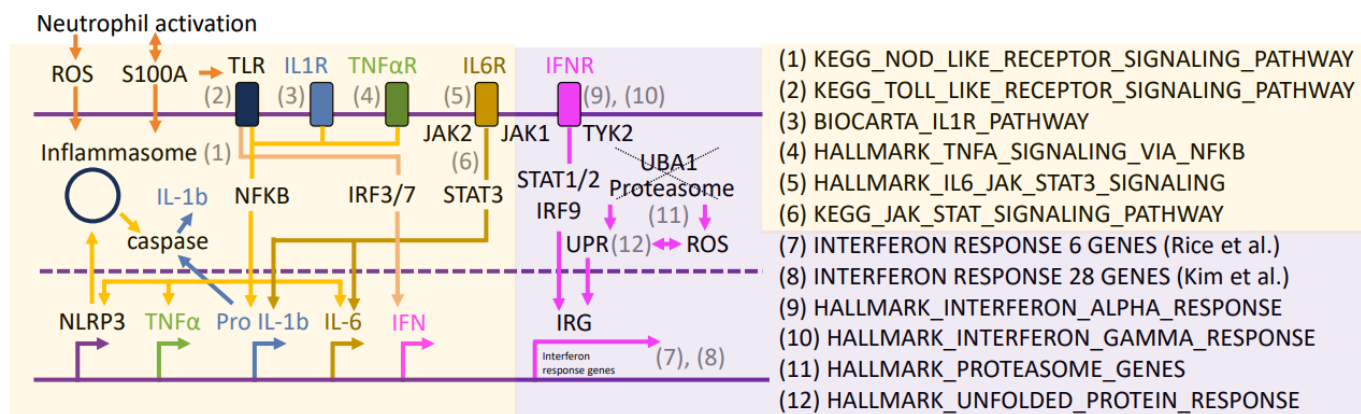

B

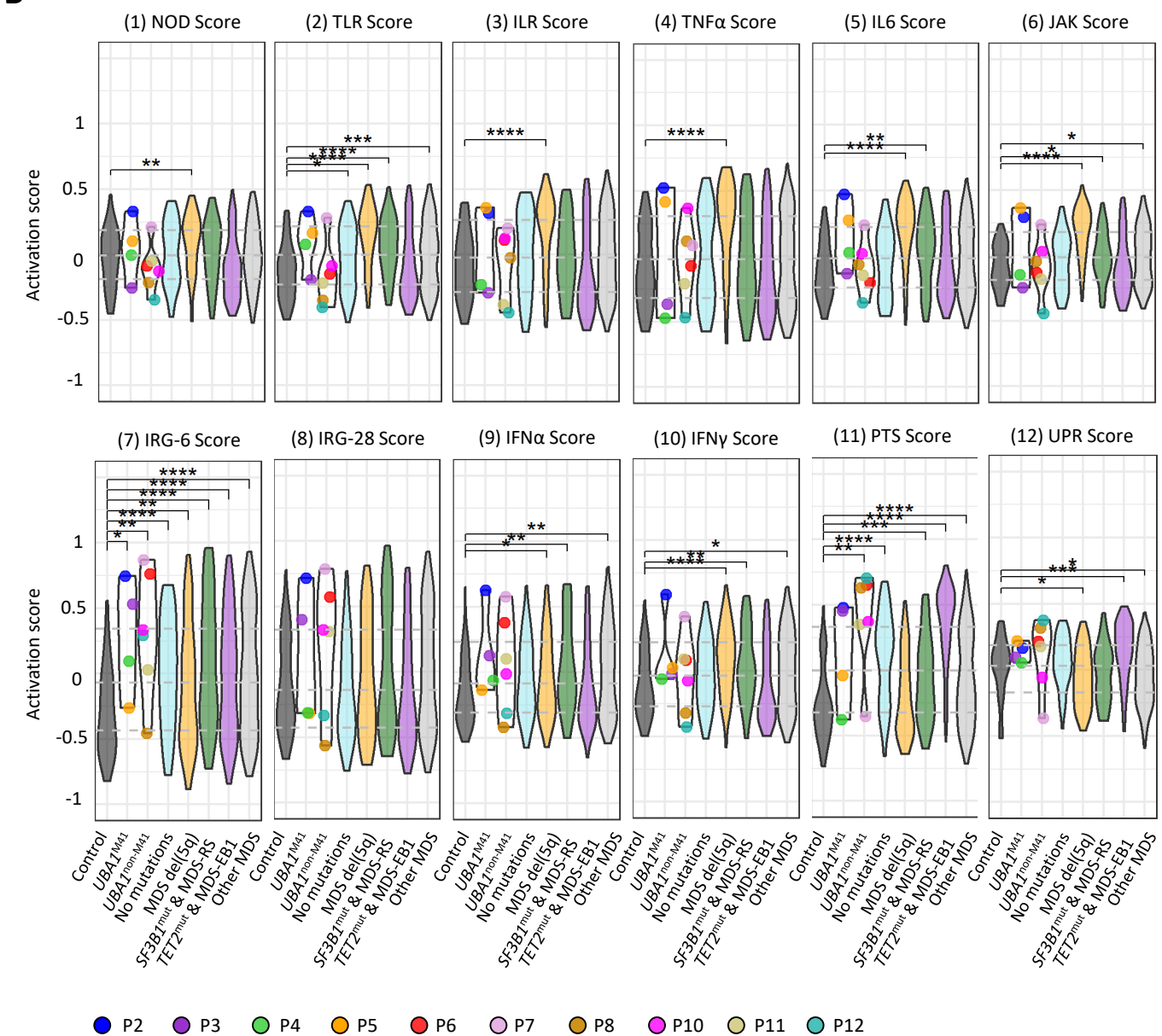

Supplement: Supplementary file 7 — Supplementary Figure 2 [file 41375_2023_1857_MOESM7_ESM.pdf]
